# Efficacy and tolerability of a low-glycemic-index ketogenic diet in Angelman syndrome: findings from the DIANE study

**DOI:** 10.1186/s13023-025-04037-z

**Published:** 2025-10-21

**Authors:** Gema Iglesias Escalera, Rosario Cazorla Calleja, María Lorenzo Ruiz, Julián Lara Herguedas, Carolina Gutiérrez Junquera, María Luz Cilleruelo Pascual, Antonio F. Caballero-Bermejo, Enriqueta Roman-Riechmann, Samuel Ignacio Pascual Pascual, Belén Ruiz-Antorán

**Affiliations:** 1 Neuropediatrics Unit, Department of Pediatrics. Hospital, Universitario Puerta de Hierro, Majadahonda, Madrid, Spain; 2 Escuela de Doctorado, Universidad Autónoma de Madrid, Madrid, Spain; 3 Pediatric Gastroenterology Unit. Department of Pediatrics. Hospital, Universitario Puerta de Hierro, Majadahonda, Madrid, Spain; 4 Faculty of Medicine, Universidad Autónoma de Madrid, Madrid, Spain; 5 Facultad de Ciencias de la Salud, Universidad Alfonso X El Sabio, Madrid, Spain; 6 Facultad de Medicina y Ciencias de la Salud, Universidad Nebrija, Madrid, Spain; 7 Department of Clinical Pharmacology, Hospital Universitario Puerta de Hierro, . C/ Joaquín Rodrigo, 1, 28222 Majadahonda, Madrid, Spain; 8 Instituto de Investigación Sanitaria Puerta de Hierro Segovia de Arana, Madrid, Spain

**Keywords:** Angelman syndrome, Diet, Ketogenic, Treatment outcome, Diet therapy, Electroencephalography, Seizures

## Abstract

**Aim:**

This study evaluates the efficacy of a low-glycemic-index diet (LGID) in improving neurodevelopmental and sleep outcomes in pediatric Angelman Syndrome (AS) patients.

**Method:**

A prospective, evaluator-blinded cohort study was conducted with 20 pediatric patients (3–16 years) diagnosed with AS. Patients were assigned to an LGID or habitual diet group and followed for 24 weeks. The primary outcome was neurodevelopmental progress measured using the Bayley Scales of Infant and Toddler Development-III. Secondary outcomes included adaptive behavior (Vineland-II), gross motor function (GMFM-88), sleep quality (actigraphy and questionnaires), seizure frequency and a 30-min awake video-EEG.

**Results:**

At 24 weeks, the LGID group showed a trend toward improvement in cognitive and language domains, although differences were not statistically significant (*p* > 0.05). Qualitative EEG improvement was observed in 44% of LGID patients versus 25% in the control group. Sleep parameters showed minor changes, with no significant differences between groups.

Interpretation: While the LGID was well-tolerated and showed trends toward neurocognitive and seizure improvements, results were not statistically significant. Further studies with larger sample sizes are needed.

## Introduction

Angelman Syndrome (AS) (OMIM 105830) is a severe neurodevelopmental disorder of genetic origin, with an estimated prevalence ranging between 1 in 12,000 and 1 in 20,000 individuals. Typical clinical features include intellectual disability with a marked delay in language development, particularly in expressive language; individuals may comprehend simple commands and demonstrate effective use of nonverbal communication. AS is characterized by a distinctive physical and behavioral phenotype, including excessive laughter, a smiling facial expression, easy initiation of social interaction, stereotyped behavior, anxiety, and hyperkinesia. Most cases are associated with epilepsy, balance and movement disorders, as well as sleep disturbances, including insomnia, nocturnal awakenings, and reduced total sleep time [1–4]. Electroencephalograms (EEG) reveal relatively specific findings (theta, delta patterns, or 3–4 Hz spike-wave discharges in posterior regions), even in the absence of clinical seizures [5, 6].

AS is caused by four distinct molecular defects in the 15q11.2-q13 imprinting region: deletion (75%), uniparental disomy (1–2%), imprinting defects (1–3%), or mutations in the UBE3A gene (5–10%). A small group of patients (10%) clinically diagnosed with AS (AS-like) exhibit no detectable anomalies in UBE3A [4, 7].

Although new gene therapies are under development, the current treatment for AS remains symptomatic. Various therapeutic approaches with the potential to treat AS are in different stages of preclinical and clinical development. Some aim to restore the deficient or dysfunctional UBE3A protein in neurons by delivering the UBE3A gene via adeno-associated viruses. Another promising approach focuses on transcribing UBE3A-ATS to "activate the dormant" paternal UBE3A gene using antisense oligonucleotides, topoisomerase inhibitors, and genome-engineering techniques. Other therapeutic targets address distinct molecular pathways known to be involved in AS pathophysiology. It is anticipated that some of these approaches could become available as disease-modifying treatments in the coming years [8, 9].

The ketogenic diet (KD) is defined as a high-fat, adequate-protein, and low-carbohydrate diet that induces ketosis. Currently, KD is established as a treatment for children and adults with refractory epilepsy. There are four major types of KD: classical, modified Atkins, medium-chain triglyceride, and low-glycemic-index diet (LGID). LGID is similar to KD but less restrictive, and it has been successfully used to reduce seizures in patients with epilepsy. AS is included among the epilepsy syndromes in which KD has shown particular benefit [10].

Several studies have reported the use of KD in AS patients for epilepsy management [10–12]; however, few studies have focused on its primary objective of improving neurocognitive and behavioral outcomes [13–17]. The effect of KD on cognition, behavior, and motor coordination has been evaluated in animal models with cognitive impairment secondary to various conditions, such as Alzheimer’s disease [14], demonstrating positive effects. In epilepsy, KD has been shown to enhance mitochondrial redox metabolism, provide neuroprotection by preventing hippocampal dysfunction, and improve cognition by enhancing visuospatial and working memory. Recent studies reveal reduced cortical and cerebellar inhibition in AS mice, along with mitochondrial dysfunction and oxidative stress, which are closely linked to memory deficits in rodents. KD may help overcome this GABA/glutamate excitatory/inhibitory imbalance, exert an inhibitory effect on neuronal excitability, stimulate mitochondrial biogenesis, prevent oxidative stress, and improve hippocampal synaptic connectivity [14, 19, 20].

These findings suggest that ketosis induced by KD in AS could be a promising strategy for improving symptoms, including epileptic seizures, motor difficulties, and severe developmental delays.

The objective of this study was to evaluate the efficacy of LGID in the treatment of AS patients, focusing on improvements in the developmental index assessed using the Bayley Scales of Infant and Toddler Development-III after 24 weeks of intervention.

## Method

### Design and participants

This prospective, evaluator-blinded, single-center cohort study compared two groups of pediatric patients aged 3 to 16 years diagnosed with Angelman Syndrome (AS): one treated with a low-glycemic-index diet (LGID) and the other following a habitual diet (control group). Both cohorts were prospectively recruited. An individualized diet was developed for each participant based on their specific requirements. The diet was designed and supervised by a nutritionist and a pediatric gastroenterology specialist, with LGID consisting of 60% fats, 20–30% proteins, and 10% carbohydrates. Ketocal® supplements were added to achieve a ketogenic ratio of 1:1. Adherence to the diet was confirmed through weekly measurements of blood and urine ketones. The follow-up period was 24 weeks.

The study was approved by the Research Ethics Committee (CEIm) of Hospital Universitario Puerta de Hierro (PI 95/21) and conducted in accordance with the Declaration of Helsinki and Good Clinical Practice guidelines. Legal guardians provided written informed consent.

### Variables

All patients included in the study were assessed at baseline and after 24 weeks using a battery of standardized tools. The primary variable was the score on the Bayley Scales of Infant and Toddler Development-III. Secondary variables included the Vineland-II Adaptive Behavior Scales (VABS-II), the Gross Motor Function Measure (GMFM), and a 30-min awake video-EEG. The EEG was used to analyze background activity, the presence of paroxysmal activity, Boyd patterns, and electrical seizures. EEGs were interpreted by neurophysiologists experienced in Angelman Syndrome (AS). The 24-week assessment was performed in a blinded and qualitative manner, with evaluators classifying results as “same,” “better,” or “worse.”

The patients’ sleep patterns were analyzed using an actigraph (Philips Actiwatch 2) worn on the ankle for 24 h a day over five consecutive days. Data were processed using Actiware software version 6.1.0. To complement sleep evaluation, parents or guardians completed questionnaires such as the Sleep Diary (parent-reported sleep quality) and the Children’s Sleep Habits Questionnaire (CSHQ). Additionally, parents subjectively evaluated their own sleep quality using specific questionnaires: the Insomnia Severity Index (ISI), with insomnia classified as > 7 points for mild, 8–14 for subclinical, 15–21 for moderate clinical, and 22–28 for severe clinical insomnia; and the Epworth Sleepiness Scale, with daytime sleepiness categorized as > 11 points for mild, 13–15 for moderate, and 16–24 for severe sleepiness. Parental stress was also assessed using the Parenting Stress Index Short Form (PSI-SF) at baseline and 24 weeks.

Communication ability was assessed using the Observer Reported Communication Ability (ORCA) scale, developed and validated at Duke University. This tool, designed specifically for individuals with neurodevelopmental disorders such as AS, was supported by an initial qualitative study involving 24 caregivers and a subsequent quantitative study involving 249 caregivers [23–25]. The scale evaluates expressive, receptive, and pragmatic communication as well as vocabulary (spoken words and symbols on assistive devices). ORCA T-scores, ranging from 26.82 to 83.24, reflect higher communication abilities with higher scores.

Comprehensive clinical follow-up included anthropometric measurements, vital signs, and laboratory parameters. Laboratory tests included complete blood count, general biochemistry, liver function, lipid profile, and measurement of blood and urine ketones.

### Statistical analysis

Descriptive analyses of categorical variables were performed using absolute and relative frequencies, while numerical variables were summarized using mean, standard deviation, median, and interquartile range (IQR).

Group comparisons were performed using the Mann–Whitney U test for numerical variables and Fisher's exact test for categorical variables, as appropriate. Pre-post intervention comparisons were conducted using the Wilcoxon signed-rank test. To compare changes between the intervention and control groups, the Mann–Whitney U test was used. The significance level was set at 0.05. All statistical analyses were conducted using R version 4.3.3.

## Results

**Fig. 1:**
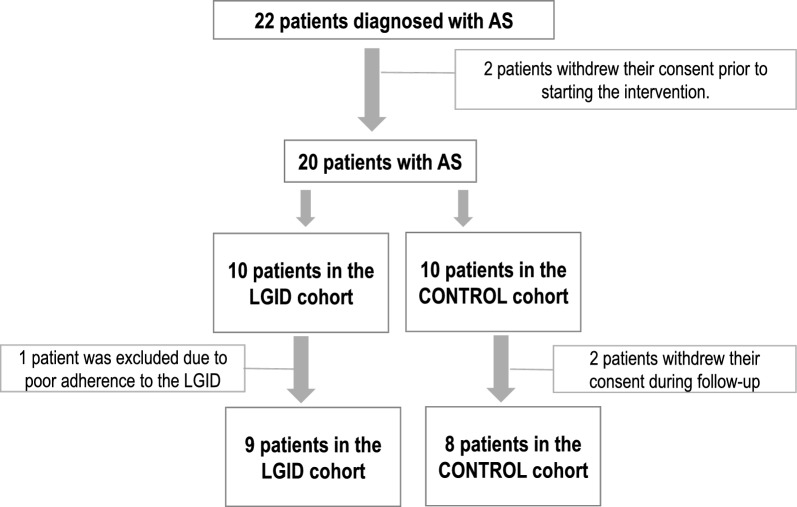
Flowchart of patient selection

During the study period, a total of 22 pediatric patients aged 3 to 16 years were recruited from a specialized Angelman Syndrome (AS) clinic. During the initial phase, two patients decided not to continue in the study for reasons related to selection, resulting in a total of 20 eligible patients. These were distributed into two cohorts: one following a low-glycemic-index diet (LGID) and the other a habitual diet (control group). Subsequently, three additional withdrawals were reported: one patient from the LGID group due to non-compliance with the dietary protocol and two patients from the control group due to decisions by legal guardians. Ultimately, the final sample consisted of 9 patients in the LGID cohort and 8 in the habitual diet cohort (control) (Fig. 1).

Baseline demographic and clinical characteristics were similar in both groups (Table 1). The sex distribution showed a higher proportion of females in the LGID cohort (56% compared to 25% in the control cohort), although this difference did not reach statistical significance (*p* = 0.335). Regarding genetic characteristics, the most common alterations were deletions (50% in the control cohort and 56% in the LGID cohort), followed by duplications and imprinting defects, the latter present only in the LGID cohort (22%).

**Table 1:** Baseline demographic and clinical characteristics in both cohorts

	Control group N = 8	LGID group N = 9
Age, years		
Mean (SD)	8.84 (2.94)	8.53 (4.65)
Median (IQR)	8.17 (6.93, 11.50)	6.99 (5.75, 11.49)
Sex, female, N (%)	2 (25)	5 (56)
Genetics, N (%)		
Deletion	4 (50)	5 (56)
Duplication	4 (50)	2 (22%)
Imprinting	0 (0)	2 (22%)
Symptoms		
Independent walking >10 steps	6 (75)	6 (67)
Epilepsy	6 (75)	9 (100)
Sleep disorder	7 (88)	7 (78)
Language disorder		
ORCA_t	8 (100)	9 (100)
Mean (SD)	48.23 (7.03)	49.71 (10.09)
Median (IQR)	49.90 (46.60, 53.0)	50.45(44.55,54.65)
Adjuvant treatments		
Antiepileptics	6 (75)	9 (100)
Hypnotics	7 (87)	7 (78)
Physiotherapy/speech therapist/occupational therapist (>2 sessions/week)	8 (100)	9 (100)

Among functional characteristics, 75% of participants in the control cohort and 67% in the LGID cohort showed the ability to walk independently for more than 10 m without assistance, with varying degrees of ataxia and/or tremor. The presence of epilepsy was high in both cohorts, reaching 100% in the LGID cohort and 75% in the control cohort, with patients receiving antiepileptic treatment (most commonly levetiracetam and valproic acid). Sleep disorders affected the majority of participants, who were treated with melatonin or aripiprazole.

All patients in both groups had severe language disorders. Linguistic function, measured through the ORCA_T index, showed equivalent means and medians in both cohorts, indicating a homogeneous profile in this domain.

One hundred percent of patients in both cohorts received physiotherapy, speech therapy, or occupational therapy at a frequency of 2–4 sessions per week, ensuring a comprehensive therapeutic approach.

**Fig. 2:**
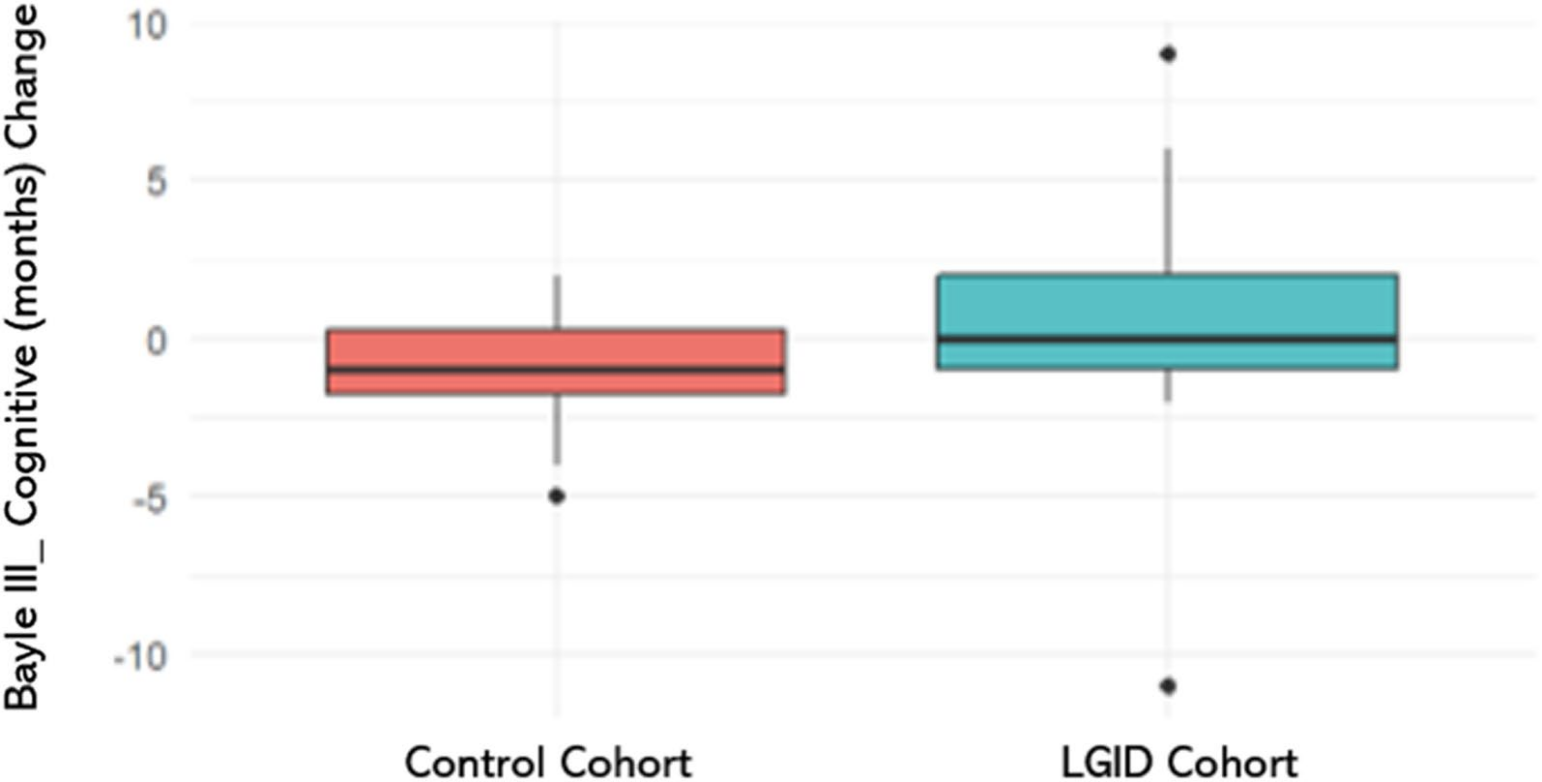
Changes in cognitive developmental age (months) after the intervention, assessed using the Bayley-III scale

Regarding the efficacy of the dietary intervention and analysis of the primary variable, the results on the Bayley-III scale showed that, at 24 weeks, the change in cognitive developmental age was 0.44 months (IQR: − 1.00, 2.00) in the LGID cohort and -1.13 months (IQR: − 1.75, 0.25) in the habitual diet cohort (control). Although the data suggest a slight improvement in the LGID cohort, the differences between cohorts did not reach statistical significance (*p* = 0.331) (Table 2; Fig. 2).

**Table 2:** Child development outcomes assessed with the Bayley-III scale at 24 weeks of intervention

	LGID cohort			Control cohort			LGID cohort	Control cohort	
Variable	Baseline (n = 9)	24 weeks (n = 9)	*p*-value ^a^	Baseline (n = 8)	24 weeks(n = 8)	*p*-value ^a^	Change after the intervention	Change after the intervention	*p*-value ^a^
Cognitive (Developmental age in months)									
Mean (SD) Median (IQR)	19.67 (7.70) 22 (14, 26)	20.11 (8.62) 15 (14, 24)	0.8	19.63 (4.96) 19 (15, 23)	18.50 (5.55) 16 (14, 22.25)	0.3	0.44 (5.55) 0 (-1, 2)	-1.13 (2.36) -1 (-1.75, 0.25)	0.331
Receptive communication (developmental age in months)									
Mean (SD) Median (IQR)	18.22 (9.28) 16 (11, 22)	21.00 (9.06) 21 (14, 29)	0.076	17.75 (6.07) 17 (13.25, 21.50)	18.00 (5.32) 21.(12.50, 22.	0.9	2.78 (4.44) 1 (0, 5)	0.25 (3.65) 1 (-1.50, 2	0.466
Expressive communication (developmental age in months)									
Mean (SD) Median (IQR)	13.44 (1.13) 14 (13, 14)	14.44 (1.13) 14 (14, 15)	0.089	14.00 (1.93) 14 (14, 14.25)	14.75 (1.75) 14 (14, 16.25)	0.4	1.00 (1.41) 1.00 (0.00, 2.00)	0.75 (1.91) 0 (0, 1.50)	0.726
Fine motor skills (developmental age in months)									
Mean (SD) Median (IQR)	19.44 (9.57) 15 (11, 27)	19.89 (10.25) 20 (11, 27)	0.8	20.75 (10.83) 17 (12.50, 26)	20.75 (9.54) 18 (15, 25)	>0.9	0.44 (5.20) 0.00 (-2.00, 0.00)	0.00 (4.75) 0.50 (-2, 3)	0.771
Gross motor skills (developmental age in months)									
Mean (SD) Median (IQR)	19.22 (7.50) 19 (13, 21)	20.56 (10.84) 20 (13, 21)	0.6	16.50 (3.25) 17.50 (16, 18.25)	18.13 (5.79) 17 (14, 19.25)	0.8	1.33 (4.50) 0 (0, 1)	1.63 (5.04) 0.50 (-2, 1.75)	0.922

^a^Mann Whitney U test

**Fig. 3:**
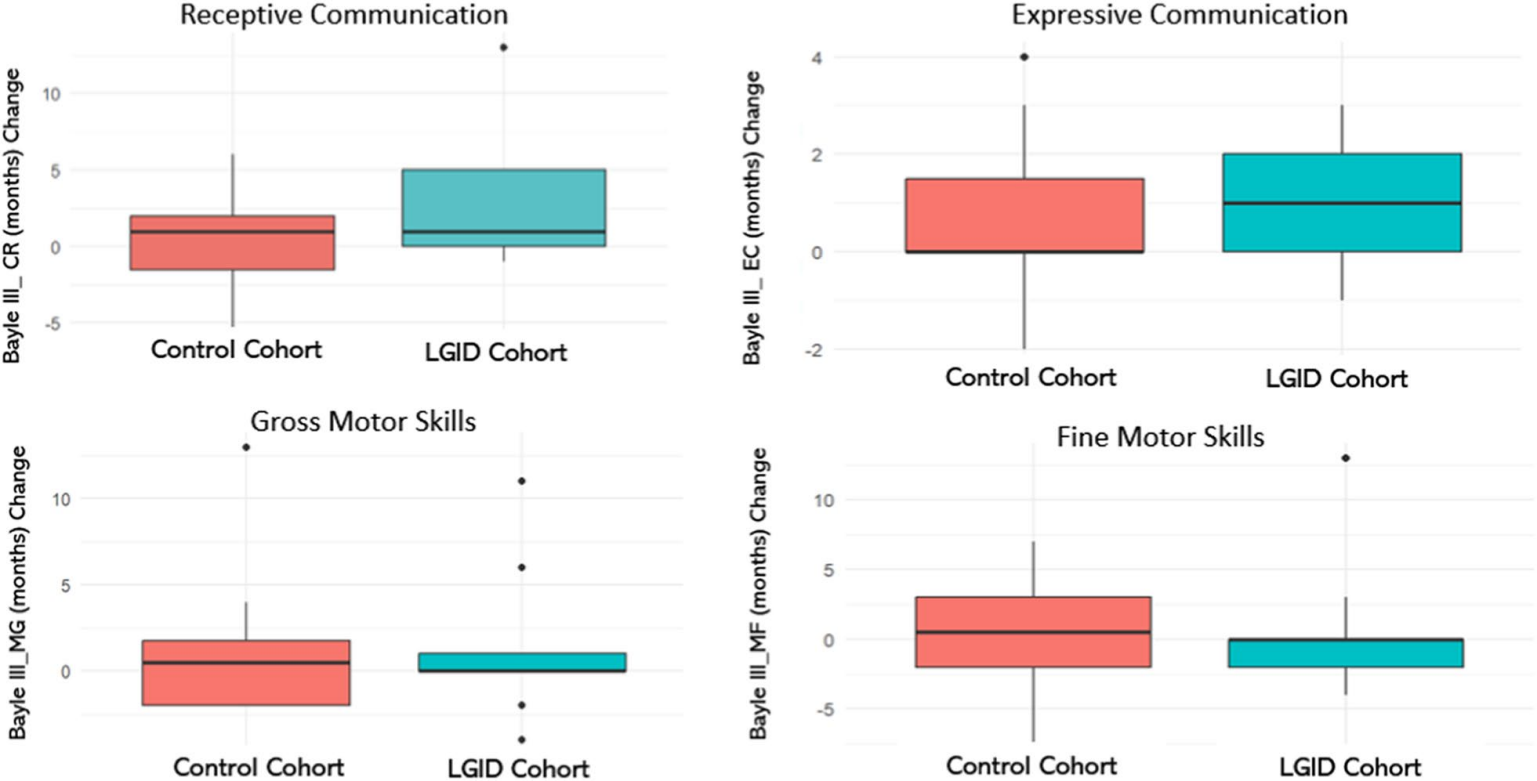
Changes in equivalent developmental age in the domains of receptive communication, expressive communication, gross motor skills, and fine motor skills (Bayley-III scale) after the intervention

In the secondary analysis of the various domains of the Bayley-III Scale evaluated at 24 weeks, including receptive communication, expressive communication, and gross and fine motor domains, no statistically significant differences were found between cohorts (Table 2; Fig. 3). Linear regression analyses adjusted for baseline values confirmed that differences in the observed changes between groups were not statistically significant in any of the evaluated domains.

Regarding secondary objectives evaluated through the Vineland-II Adaptive Behavior Scale (VABS-II), a slight improvement was observed in the communication domain in the LGID group compared to the control group (mean improvement: 2.33 vs. 1.35; *p* = 0.626). However, in the daily living skills domain, progression was more favorable in the control group compared to the LGID group. In the other domains evaluated, positive changes in the LGID group were minimal, and none reached statistical significance. Evaluation using the Gross Motor Function Measure-88 (GMFM-88) showed a greater global score improvement at 24 weeks in the LGID group compared to the control group (mean improvement: 8.11 vs. 3.13; *p* = 0.191). Although this trend suggests a potential benefit of the LGID intervention, the differences were not statistically significant (Table 3).

Regarding epilepsy, 75% of patients in the control group and 100% of patients in the LGID group had a confirmed diagnosis of epilepsy. Baseline EEGs were abnormal in all cases, showing typical electrophysiological characteristics of Angelman Syndrome (AS). During the study, one patient from each group (LGID and control) experienced epileptic seizures. In the control group, one patient required hospitalization due to non-convulsive status epilepticus without a clear trigger, although sleep disturbances were reported in the preceding days. EEG recordings showed no deterioration at 24 weeks compared to baseline in any patients. Qualitative EEG improvement was observed in 44% of patients in the LGID group compared to 25% in the control group, although these differences were not statistically significant.

Regarding the impact of the dietary intervention on sleep characteristics in patients with Angelman Syndrome and the stress and sleep of their parents, the results showed minimal changes in several evaluated parameters, and no statistically significant differences were reached in any of the analyzed variables. In patients, sleep characteristics assessed through the Children’s Sleep Habits Questionnaire (CSHQ) and actigraphy indicated that the total CSHQ score slightly decreased in the LGID cohort, with a mean change of − 0.17 points (IQR: − 3.25, 2.25), while it increased by 0.80 points (IQR: − 3.00, 4.00) in the habitual diet cohort (*p* = 0.714). Total sleep time showed an average increase of 29.9 min (IQR: − 30.50, 80.00) in the LGID group compared to a decrease of − 11.00 min (IQR: − 32.75, 23.25) in the habitual diet group (*p* = 0.412). No statistically significant differences were found in other parameters, such as sleep latency, number of awakenings, sleep efficiency, or wake time after sleep onset (dTIS).

Regarding the impact on parents’ sleep and stress, the Parenting Stress Index Short Form (PSI-SF) showed a significant reduction within the LGID cohort, with a mean change of − 13.25 points (IQR: − 19.25, − 6.00), while the habitual diet cohort showed a mean change of − 5.20 points (IQR: − 13.00, 8.00). However, differences between cohorts did not reach statistical significance (*p* = 0.557). Other parameters, such as the Insomnia Severity Index (ISI), Epworth Sleepiness Scale, and perception of sleep quality, showed no significant differences between cohorts, with minimal variations in both (Table 4).

Regarding diet tolerance, no adverse events occurred in either treatment cohort. The LGID was well tolerated, with normal clinical and laboratory controls performed at both 12 and 24 weeks.

## Discussion

Our study, which included pediatric patients with Angelman Syndrome (AS), did not demonstrate that the low-glycemic-index diet (LGID) produces clinical improvement in any of the neurodevelopmental domains assessed using the Bayley Scales of Infant and Toddler Development-III.

Although various publications explore the use of the ketogenic diet (KD) in patients with AS, most of these studies focus on its efficacy for epilepsy control. However, there are few studies where the primary objective is to analyze the potential effects of KD on neurocognitive and behavioral development in this population. Previous research has reported that KD has positive cognitive and behavioral effects in pediatric patients with epilepsy, regardless of seizure control or the number of concomitant antiepileptic drugs [11, 15–17]. These benefits include improvements in alertness, attention, reciprocal social interaction, mood, sustained attention, receptive vocabulary, and information processing speed. Furthermore, various reviews have reported subjective data from parents describing their children as “more awake” and “more attentive” after starting the diet [17].

In the specific population with AS, Grocott et al. [18] retrospectively reviewed 23 patients treated with LGID and found that most achieved improved seizure control: 22% remained seizure-free, 43% experienced seizures only in specific contexts such as illness or non-convulsive status, and 30% showed a significant reduction in seizure frequency. Additionally, Thibert et al. [13] conducted a prospective study on the efficacy and tolerability of LGID in AS patients, reporting a reduction in seizure frequency in all patients, a reduction greater than 80% in five of them, and generalized improvements in EEG patterns. This study also reported a subjective perception of neurodevelopmental improvement from parents, although only some of these improvements were statistically significant in neuropsychological assessments.

Our results align with these findings. In our study, we observed qualitative improvement in the EEGs of patients treated with LGID compared to the habitual diet group after 24 weeks of intervention (44% improvement in the LGID group versus 25% in the habitual diet group). Moreover, in the LGID group, only 11% of patients experienced clusters of epileptic seizures, compared to 25% in the habitual diet group, which included one case of non-convulsive status epilepticus.

Although no statistically significant cognitive differences were achieved, a trend toward improvement was observed in receptive language, expressive language, and communication domains in the LGID group. Similarly, subjective parental perception reflected a global improvement in neurodevelopment in most cases. This outcome aligns with previous observations and reinforces the hypothesis that LGID may have neurocognitive benefits in patients with AS. In fact, five of the nine patients in the LGID group chose to continue the diet after completing the study, suggesting a positive impact perceived by families.

Sleep plays a fundamental role in the development and maintenance of memory and learning. Improving sleep quality and structure can significantly enhance sustained attention and memory in children. In patients with Angelman Syndrome (AS), it is estimated that approximately 80% experience moderate to severe sleep disturbances. Pelc et al. [26] conducted a clinical review in a small group of AS patients and described specific sleep characteristics, such as reduced total sleep duration, increased sleep-onset latency, altered sleep architecture, frequent nighttime awakenings, and reduced REM phase. Similarly, Spruyt et al. [27] conducted a systematic review and meta-analysis of 14 heterogeneous studies, mostly observational, and concluded that characteristic sleep problems in AS include reduced total sleep time, increased latency, frequent awakenings, and reduced sleep efficiency.

Additionally, Miano et al. [29] evaluated 10 children with AS using polysomnography and compared them with a control group of patients with intellectual disabilities with or without epilepsy. The results showed a significant increase in sleep state transitions, four times more frequent awakenings, and a 50% reduction in time spent in the deepest stage of sleep (NREM). This suggests considerably reduced sleep quality and lower sleep efficiency in AS patients.

Our results are consistent with these observations, as patients in our series showed reduced total sleep time, increased latency, frequent awakenings, and decreased sleep efficiency.

Pasca et al. [30] reviewed the effects of the ketogenic diet (KD) on sleep in neurological conditions such as autism spectrum disorders, epilepsy, and migraines, finding improvements in overall sleep quality, sleep-onset latency, reduction of nighttime awakenings, improved daytime sleepiness, and increased REM sleep. In our study, although a trend toward improvement in sleep quality was observed in the LGID group compared to the habitual diet group, these data were not statistically significant, preventing the assertion that LGID alone improves sleep structure.

It is important to note the limitations of this study. These include the small sample size, inherent to rare diseases such as Angelman syndrome, which limits statistical power. Additionally, the 24-week follow-up may have been insufficient to detect clinically meaningful changes in neurodevelopmental parameters, which often require longer durations to reach statistical significance. Developmental age equivalents were used as the primary outcome measure instead of more sensitive psychometric scores such as the Person Ability Score (PAS). Although this choice aligned with standard practice in 2021, it may have limited the detection of subtle changes in this population. Similarly, the use of standard scores from the VABS-II may have lacked sensitivity to small variations over time, particularly in individuals with profound impairment. As with the Bayley-III, this approach was consistent with prevailing clinical guidelines and research practices in Angelman syndrome; however, more sensitive metrics such as PAS could not be derived due to the unavailability of item-level conversion tools. Finally, some missing data resulted from incomplete caregiver diaries, likely due to the emotional and time burden of caregiving, as well as issues with the use of the Actiwatch by some children, which limits the reliability of the data.

In conclusion, although a trend toward greater evolutionary improvement was observed in the LGID group compared to the habitual diet group, these differences were not statistically significant in the various domains evaluated using the Bayley Scales of Infant and Toddler Development-III and cannot be solely attributed to the low-glycemic-index ketogenic diet. However, global improvements were identified in several variables evaluated at six months in the LGID group, and no serious adverse reactions attributable to the diet were reported.

These preliminary results do not support recommending the low-glycemic-index ketogenic diet as a generalized treatment for cognitive improvement in AS patients. Further studies with larger sample sizes and robust designs are needed to evaluate the potential impact of this intervention in this population.

## Data Availability

Not applicable.
